# Improving the electrochemical performances using a V-doped Ni-rich NCM cathode

**DOI:** 10.1038/s41598-019-45556-7

**Published:** 2019-06-20

**Authors:** Seoung-Ju Sim, Seung-Hwan Lee, Bong-Soo Jin, Hyun-Soo Kim

**Affiliations:** Next-Generation Battery Research Center, Korea Electrotechnology Research Institute, Changwon, 641-120 South Korea

**Keywords:** Batteries, Batteries

## Abstract

Ni-rich layered LiNi_0.84_Co_0.10_Mn_0.06_O_2_ cathode material was modified by doping with vanadium to enhance the electrochemical performances. The XRD, FESEM and XPS analyses were indicated that the vanadium is successfully doped in the crystal lattice of LiNi_0.84_Co_0.10_Mn_0.06_O_2_ with high crystallinity. 0.05 mol% vanadium doped LiNi_0.84_Co_0.10_Mn_0.06_O_2_ exhibits superior initial discharge capacity of 204.4 mAh g^−1^, cycling retention of 88.1% after 80 cycles and rate capability of 86.2% at 2 C compared to those of pristine sample. It can be inferred that the vanadium doping can stabilize the crystal structure and improve the lithium-ion kinetics of the layered cathode materials.

## Introduction

Lithium-ion batteries (LIBs) are being widely used for portable device, uninterruptible power supplies (UPS), hybrid electric vehicles (HEVs), electric vehicles (EVs) and so on. The recent demand for LIBs has been considerably increasing and the research for LIBs focuses on high capacity, long cycle life and high-rate capability. Recently, Ni-rich layered oxide has received a lot of attention due to their higher specific capacity, lower cost and lower toxic than other cathode materials^1^. However, the long-term cycling stability is proportionally deteriorated with increasing Ni contents. It can be caused by surface side reactions, phase transitions during charge-discharge, the reduction of Ni^4+^ and oxygen release, resulting in deterioration the crystal structure. This phenomenon suffers a decrease in electrochemical performance, and hinder commercialization^2,3^.

Many efforts have been made to solve these problems and modify cathodes using coatings^4^, dopants^5^, core-shell^6^, concentration-gradient structures^7^ and single crystalline materials^8,9^. Among them, doping is an effective strategy to improve stability of layered structure because the dopant ions help suppression of the phase transition and the voltage fading. So far, lots of single dopants have been explored such as Al^10^, Ti^11^, Zr^12^, Mg^13^, Mo^14^, Na^15^, Sn^16^, B^17^, Fe^18^ and Cr^19^ to improve the electrochemical performance of layered cathode materials. Vanadium was also studied as one of the dopant by many researchers because vanadium can take various valence state, leading to the stability of the crystal and increase of Li-ion diffusion coefficient^20–23^. These can significantly improve the capacity, coulombic efficiency, cycleability and rate capability. To the best of our knowledge, the effect of the vanadium doped Ni-rich NCM have still not been studied.

Therefore, in this paper, we synthesized well-crystallized V-doped Ni-rich LiNi_0.84_Co_0.10_Mn_0.06_O_2_ (hereafter V-doped NCM) by solid state method to stabilize the crystal structure and deliver superior electrochemical performances.

## Experimental Section

**Figure 1:**
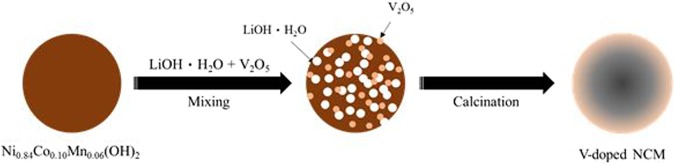
Schematic illustration of the synthesis process of V-doped NCM.

For the synthesis of LiNi_0.84_Co_0.10_Mn_0.06_O_2_, the Ni_0.84_Co_0.10_Mn_0.06_(OH)_2_ precursor was prepared by a co-precipitation method. An aqueous solution of NiSO_4_·6H_2_O, CoSO_4_·7H_2_O and MnSO_4_·H_2_O was used as starting materials. Simultaneously, a NaOH solution and NH_4_OH solution as a chelating agent were also used. The prepared spherical Ni_0.84_Co_0.10_Mn_0.06_(OH)_2_ precursor was mixed with LiOH·H_2_O at a molar ratio of 1: 1.05 and V_2_O_5_ powder as a vanadium source with the molar ratios of 0, 0.005, 0.01 and 0.02 mol%. After that, the powders were calcined at 500 °C for 5 h and 760 °C for 15 h in air. V-doped NCM was prepared as illustrated in Fig. 1.

The crystal structure of the materials was characterized by X-ray diffraction (XRD, X-pert PRO MPD, Philips, Cu Kα), in the 2θ range of 10°–80°. The morphology of the samples was observed by field emission scanning electron microscopy (FESEM, S-4800, HITACHI) and elemental mappings of the samples were measured by an energy dispersive X-ray detector (EDX, X-maxN, HORIBA). The chemical state of transition was conducted by X-ray photoelectron spectroscopy (XPS, VG SCIENTIFIC, ESCALAB 250, Al Kα radiation).

For the electrochemical performance, the cathode electrode was prepared by mixing 96 wt% active material, 2 wt% super P and 2 wt% polyvinylidene fluoride (PVDF) binder. The mixed slurry was coated on Al foil (16 μm in thickness) and dried at 100 °C for 10 h in a vacuum oven. After that, the electrode was punched into disks and then dried at 120 °C for 10 h. A 2032 coin cells were fabricated with pristine and V-doped NCM as a cathode and lithium foil (500 μm in thickness) as an anode. A polyethylene (PE, 20 μm in thickness) was used as a separator and 1 M LiPF_6_ in a mixed solution containing ethylene carbonate (EC)/dimethyl carbonate (DMC)/ethylmethyl carbonate (EMC) (1:1:1, v/v/v) was employed as electrolyte. The assembly of all coin cells was carried out in an argon-filled glove box.

Charge-discharge test was carried out galvanostatically under the voltage range of 3.0–4.3 V and various current density using electrochemical equipment (TOSCAT-3100, Toyo system) at 25 °C. Cyclic voltammetry (CV) of the samples was carried out by multi potentiostat (VSP300, Bio-Logic) between 3.0 and 4.3 V at a scan rate of 0.1 mV s^−1^. The electrochemical impedance spectroscopy (EIS) measurement was conducted with a VSP300 impedance analyzer using the frequency range of 1 MHz to 10 mHz with 5 mV amplitude.

## Results and Discussion

**Figure 2:**
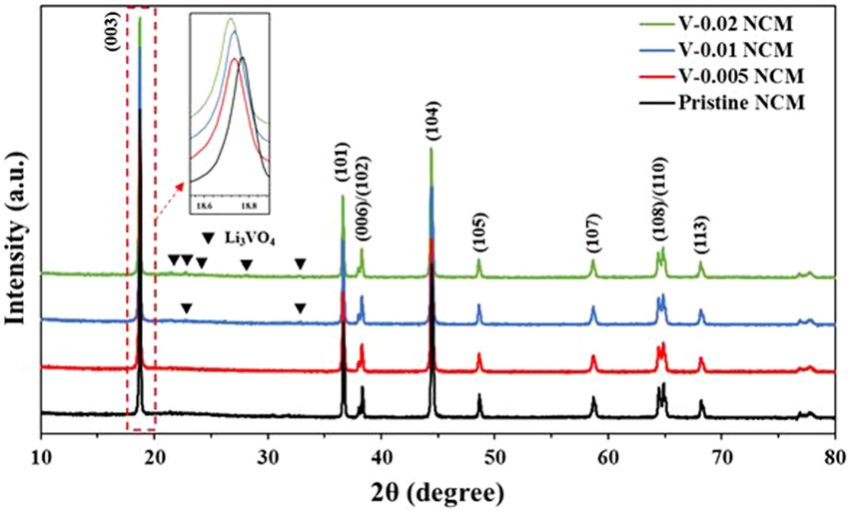
XRD patterns of pristine and V-doped NCM samples.

The XRD patterns of pristine and V-doped NCM are shown in Fig. 2. The patterns are indexed based on a layered hexagonal α-NaFeO_2_ structure with the space group R-3m^24^. It can be inferred that V-doped NCM maintains the crystal structure of pristine NCM since XRD patterns of V-doped NCM are similar to that of pristine NCM due to low doping concentration^25,26^. The V-doped NCM has a clear peak splitting of the (006)/(102) and (108)/(110), indicating well-ordered layered structure^27^. The (003) peak shifts to lower angle with increasing V content by Bragg’s law (nλ = 2dsinθ), indicating vanadium was successfully incorporated into the NCM cathode. It can be inferred that the V^5+^ ions, having larger ionic radius (0.59 Å) than those of Ni^2+^ (0.56 Å), Co^3+^ (0.54 Å) and Mn^4+^ (0.53 Å), were substituted into the lattice of the NCM^28^. It can also be explained by charge compensation that dopant can change the valence states of transitional metal with different ionic radii via vanadium doping^29^. Therefore, the lattice parameters increase linearly with an increase in doping concentration, enabling smooth and fast kinetics of Li ions^30^. The I_(003)/(104)_ ratio indirectly means the degree of cation mixing in the layered structure, resulting from similar ionic radius of Li^+^ (0.76 Å) and Ni^2+^ (0.69 Å)^31^. There is a little difference in the I_(003)/(104)_ ratios and the ratios of pristine, 0.005 mol% (V-0.005 NCM), 0.01 mol% (V-0.01 NCM) and 0.02 mol% (V-0.02 NCM) doped NCM were 1.31, 1.35, 1.29 and 1.25, respectively. The V-0.005 NCM shows the highest I_(003)/(104)_ ratio of 1.35, indicating the low cation mixing with lithium ions at the 3a site, transition-metal ions at the 3b site^32^. We can assume that the cation mixing is decreased by replacing Ni in NCM with V, beneficial to Li ion diffusion. However, excessive V doping above 0.01 mol% shows a lower I_(003)/(104)_ ratio, indicating high Li^+^/Ni^2+^ cation mixing, since it can lead to deformation of the NCM original structure^30^. Therefore, we can infer that suitable vanadium substitution can reduce the Li/Ni cation mixing with well-ordered layered structure.

**Figure 3:**
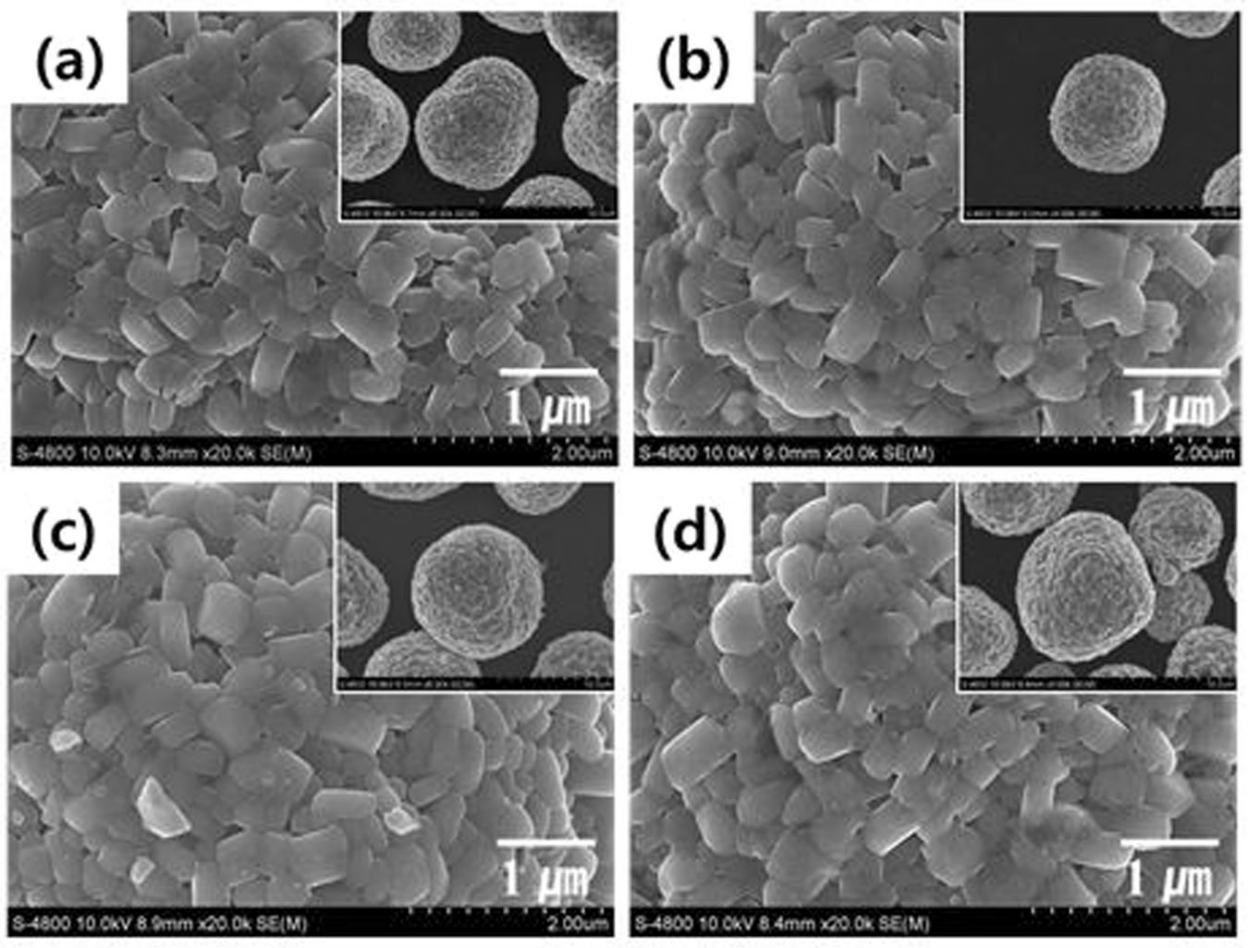
FESEM images of pristine and V-doped NCM samples: (**a**) pristine, (**b**) V-0.005 M, (**c**) V-0.01 M, and (**d**) V-0.02 M.

Figure 3 shows the FESEM images of the pristine and V-doped NCM samples. All powders show a similar spherical morphology and no great difference between the samples. The spherical samples are composed of numerous primary particles. The primary particles have similar size (500 nm) regardless of the vanadium doping level. We can infer that vanadium doping do not affect the morphology and size of NCM materials. The porous structure of spherical NCM enables to rapid Li ion kinetics, derived from sufficiently soaked liquid electrolyte^33^.

**Figure 4:**
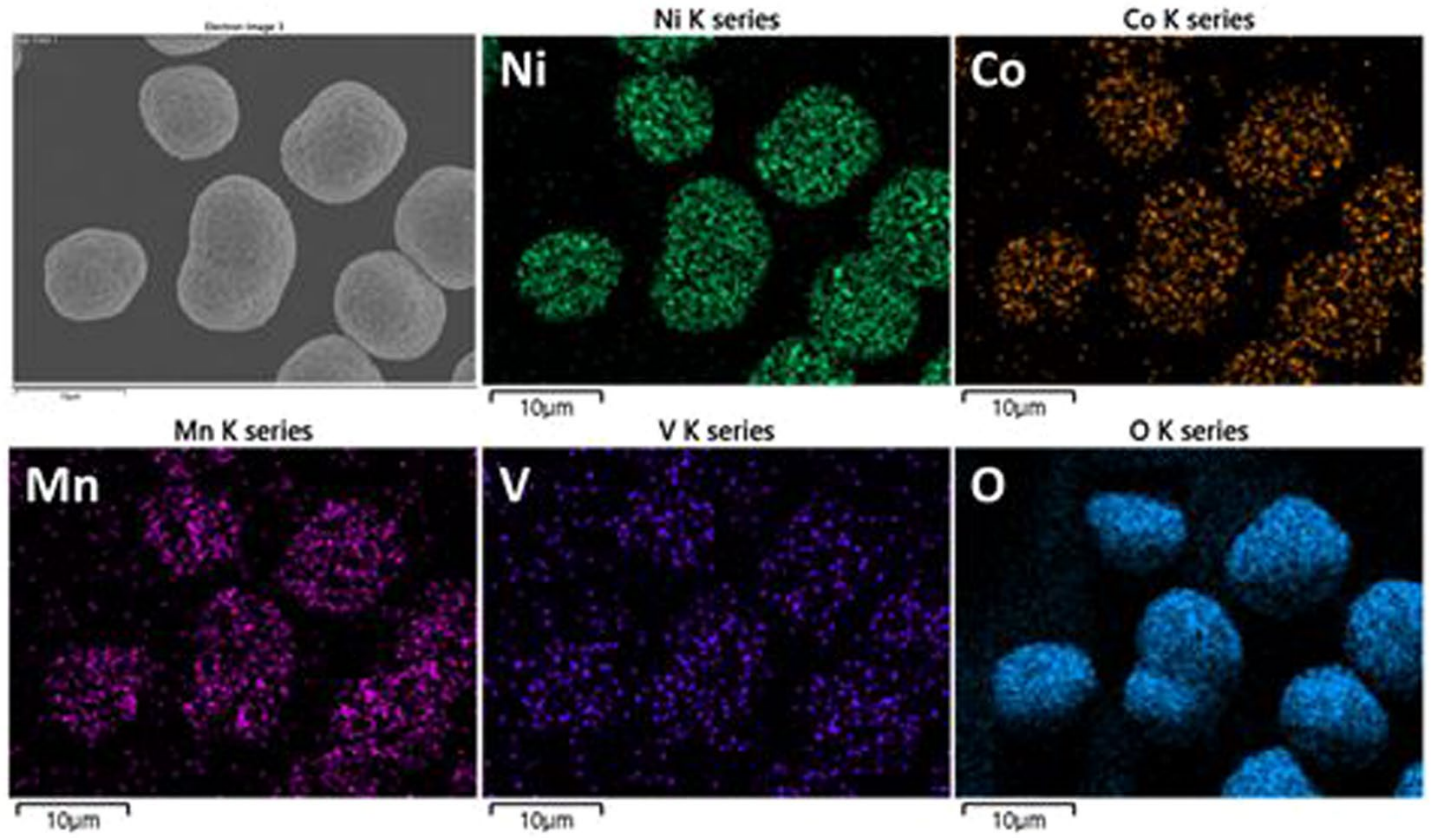
EDX mapping images of V-doped NCM sample.

Figure 4 shows EDX mapping of V-0.005 NCM to confirm the presence of doped vanadium within the NCM. It can be seen that the elements of Ni, Co, Mn, V and O were uniformly distributed in the sample since there is no agglomeration and void region in the mapping of vanadium.

**Figure 5:**
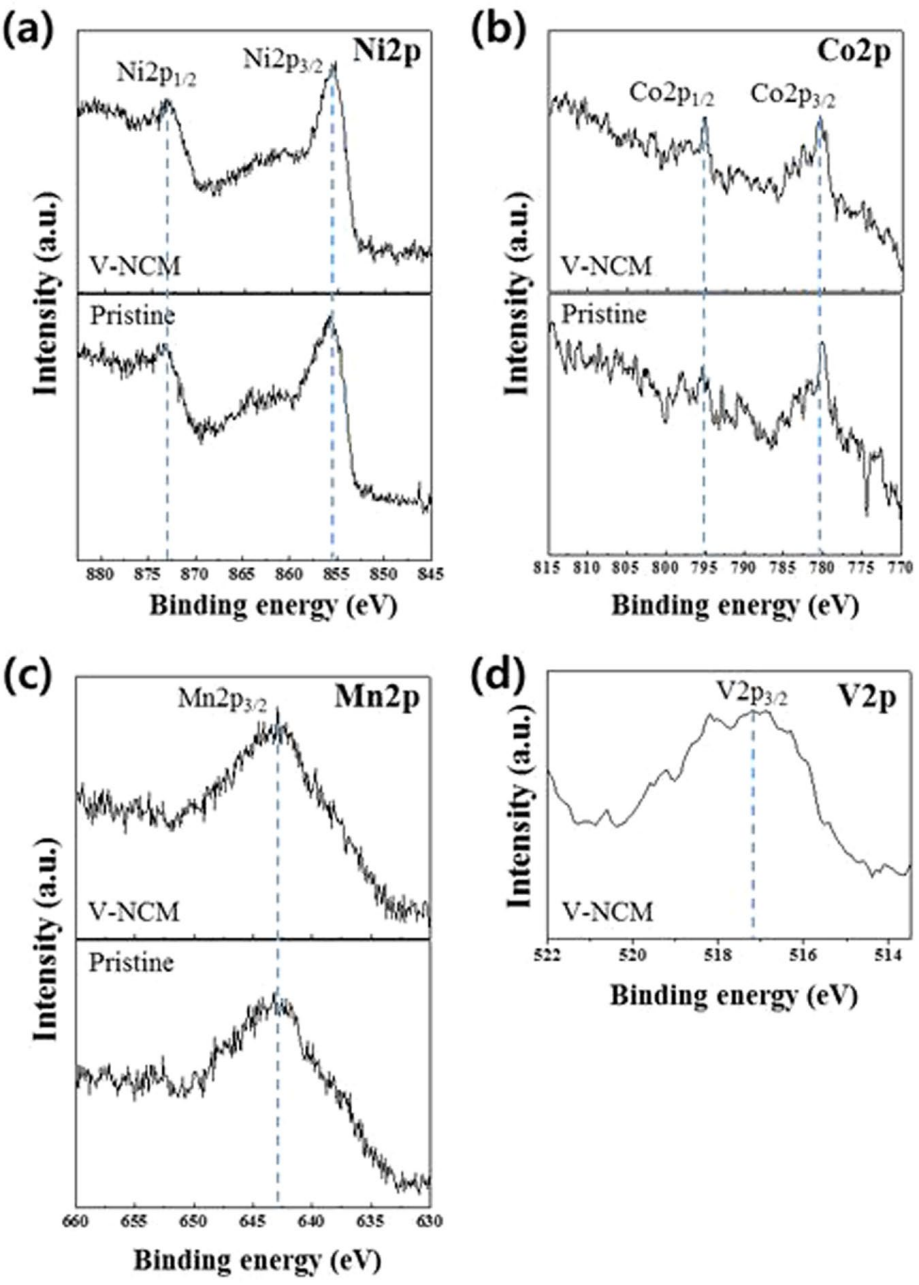
XPS spectra of (**a**) Ni2p, (**b**) Co2p, (**c**) Mn2p and (**d**) V2p for pristine and V-doped NCM samples.

XPS analysis was performed to gain further information of composition and the chemical state of V-doped NCM. The XPS spectra of pristine and V-0.005 NCM are shown in Fig. 5 to confirm the valance states of transition metal elements (Ni, Co, Mn and V). The Ni, Co and Mn elements have almost similar binding energy before and after V-doped NCM. The Ni2p spectra of samples are characterized by Ni2p_3/2_ and Ni2p_1/2_. The Ni2p_3/2_ peak appears at 855.8 eV, which means a high oxidation state^34,35^. The binding energies of Co2p_3/2_, Co2p_1/2_ appear at 781.8 eV, 795.4 eV, indicating the dominant Co^3+^ cation. The Mn spectrum has the binding energy of 644.3 eV for the Mn2p_2/3_, indicating Mn^4+^ cation^36^. It agrees with the previous report for the NCM cathode materials^37^. Although no peaks corresponding to dopant vanadium are observed at the XRD (Fig. 2) due to its small amount, it was confirmed by XPS analysis. The binding energy value of Vp_3/2_ was detected at 517.2 eV, which implies that the valance of vanadium is a high oxidation state of V^5+^ ^38^. From these results, we can confirm that the obtained sample is V-doped NCM^39^. The charge compensation derived from vanadium doping can increase the electronic conductivity of the NCM^29^.

**Figure 6:**
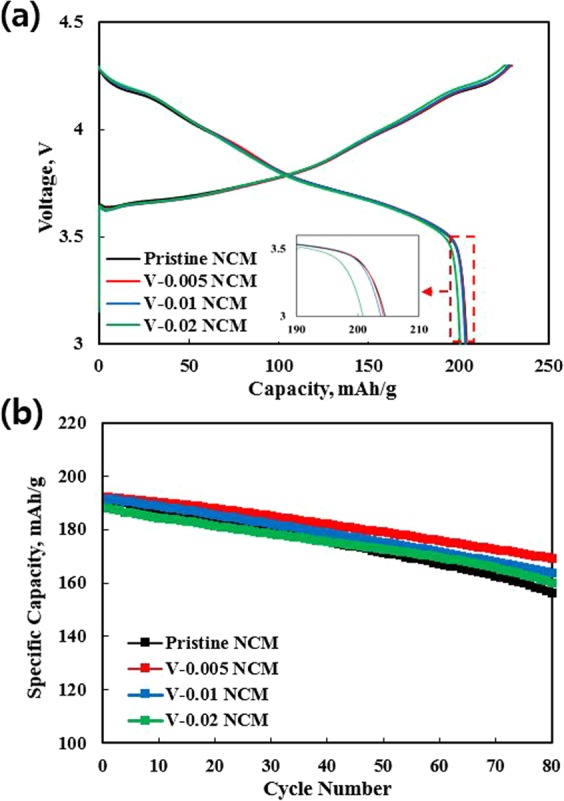
Initial charge-discharge curves at 0.1 C (**a**) and cycle performance at 0.5 C (**b**) of pristine and V-doped NCM samples.

For electrochemical tests, the loading level of the electrode was adjusted about 14.5 mg/cm^2^ to meet similar condition of commercial cathode electrode. The initial charge-discharge curves (Fig. 6(a)) and cycling performance (Fig. 6(b)) of pristine and V-doped NCM samples were measured at the rate of 0.1 C and 0.5 C (202 mAh g^−1^ at 0.1 C), respectively in a potential range of 3.0–4.3 V at 25 °C. All the samples displayed typical charge-discharge profiles of Ni-rich NCM materials^40^. The pristine delivered the highest discharge capacity (204.6 mAh g^−1^) and coulombic efficiency (89.6%). The initial discharge capacity and columbic efficiency slightly decreased with increasing the V content. It can be ascribed to the partially substituted electrochemically inactive V ions, occupying the electrochemically active transition-metal site (3b site), as reported earlier by Zhu *et al*.^22^. There was only a very slight difference between pristine and V-0.005 NCM. It was reported that the most of vanadium can enter the transition-metal site in the crystal lattice^22,28^. The small amount of vanadium dopant remaining in the NCM generates Li_3_VO_4_ impurity at the surface, resulting in low electrochemical activity due to reduced active materials, as shown in Fig. 2(b)^22^. However, V-doped NCM samples showed the superior cycle stability than that of pristine, as shown in Fig. 6(b). Among the V-doped NCM samples, V-0.005 NCM exhibits the best cycle retention. The capacity retention of V-0.005 NCM was 88.1% after 80 cycles. It can be ascribed to the bonding energies between vanadium and oxygen is stronger than that of transition metal (Ni, Co and Mn)-oxygen^22^. In addition, less Ni^2+^ ions occupy the Li^+^ sites for the NCM, leading to the NCM structure more stable. Thus, substituted vanadium ions into transition metal sites contribute to the structural stability during long-term cycling. The results are summarized in Table 1.

**Table 1:** Electrochemical results of pristine and V-doped NCM samples.

Sample	Initial charge capacity (mAh g^−1^)	Initial discharge capacity (mAh g^−1^)	Initial coulombic efficiency (%)	Capacity retention after 80 cycles (%)
Pristine	228.3	204.6	89.6	81.7
V-0.005	229.5	204.4	89.1	88.1
V-0.01	228.6	203.9	89.2	85.5
V-0.02	226.1	200.9	88.8	85.2

**Figure 7:**
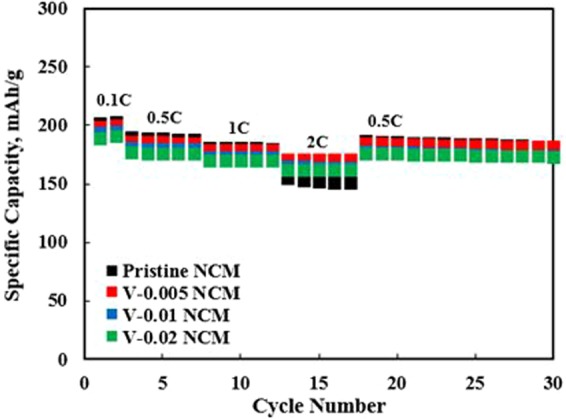
Rate capability of pristine and V-doped NCM samples.

Figure 7 shows the rate performance of the pristine and V-doped NCM samples at different discharge current rates from 0.1 C to 2 C. The capacity of pristine NCM displayed slightly higher compared to V-doped NCM samples at low current rate from 0.1 C to 1 C. However, we can confirm that the large difference in the capacity of pristine and V-doped NCM was found at the high current density of 2 C. Although the capacity of pristine NCM is slightly higher compared to V-doped NCM, it drops off significantly with increasing current density, especially at high current density of 2 C. On the other hand, V-doped NCM samples maintained high discharge capacities at 2 C. It means that that vanadium doping is favorable to excellent rate capability derived from increasing the electronic conductivity and lattice parameters, resulting in rapid Li ion kinetics^22,41^.

**Figure 8:**
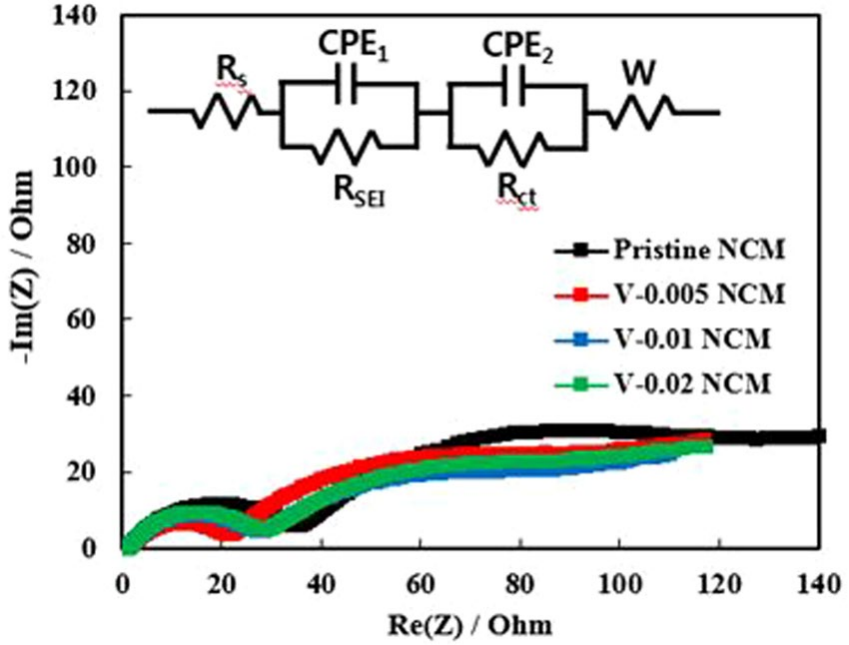
Nyquist plots of pristine and V-doped NCM samples after 80 cycles.

To investigate the effect of the vanadium doping on the electrolyte and NCM interfacial resistance, electrochemical impedance spectroscopy (EIS) were measured after 80 cycles, as shown in Fig. 8. In general, Nyquist plot consist of two semi semicircles and one slope. The electrolyte resistance (R_s_) in the high frequency is not considered because the same electrolyte was used in this paper^42^. The semicircle at high frequency is related to resistance of solid electrolyte interface (R_SEI_) and the semicircle in the middle frequency represents the charge transfer resistance (R_ct_) at the interface between the electrode and electrolyte. The straight line with the slope of 45° at low frequencies, corresponding to the Warburg impedance, is related to Li^+^ diffusion in the bulk electrode^43^. We can observe that V-doped NCM exhibited lower R_SEI_ and R_ct_ than those of pristine NCM, as shown in Table 2. The low R_SEI_ value of V-doped NCM is closely related to the stable surface chemistry in NCM since the total bonding energy of metal-oxygen on the surface alleviates the surface degradation, as mentioned in Fig. 5. More importantly, it was reported that the R_ct_ is the key factor for the cathode impedance of cell, determining the electrochemical activity^44^. As shown EIS spectra, the V-0.005 NCM represented the lowest R_ct_. Therefore, EIS result indicates that vanadium substitution can effectively suppress the increase in R_SEI_ and R_ct_ which could facilitate efficient lithium ion intercalation, resulting in superior rate capability and cyclability.

**Table 2:** R_SEI_ and R_ct_ values of pristine and V-doped NCM samples after 80 cycles.

	R_SEI_(Ω)	R_ct_(Ω)
Pristine	35.838	110.58
V-0.005	20.826	85.55
V-0.01	26.660	81.43
V-0.02	27.211	94.13

**Figure 9:**
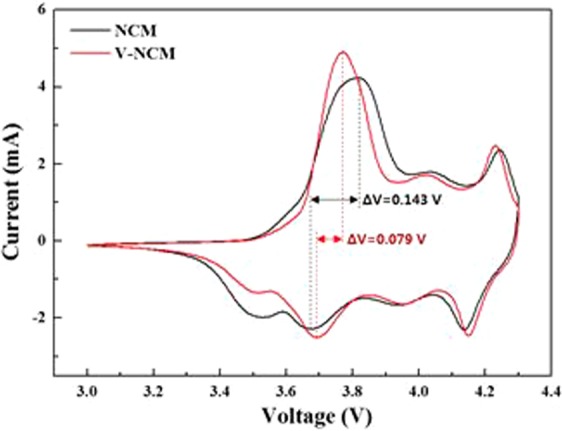
The cyclic voltammetry of pristine and V-doped NCM samples.

To demonstrate the reason for the improved performances of V-0.005 NCM, CV measurements were conducted. Figure 9 shows the initial CV curves of pristine and V-0.005 NCM between 3.0 and 4.3 V at a scan rate of 0.1 mV s^−1^. The oxidation/reduction peaks of pristine and V-0.005 NCM were observed around 3.82 V/3.67 V and 3.77 V/3.69 V, respectively, corresponding to Ni^2+^/Ni^4+^ ^21^. Also, the redox peaks were observed around 4.25 V/4.14 V and 4.23 V/4.15 V, respectively, which are corresponding to Co^3+^/Co^4+^ ^40,45^. As can be seen in Fig. 9, the potential difference (ΔV) between anodic and cathodic peaks of V-0.005 NCM, indicating polarization, is smaller (0.079 V) than pristine (0.143 V). It suggests that V-0.005 NCM has a better electrochemical reversibility during the charge/discharge process^46–48^. These low R_ct_ and polarization values ensure slow capacity fading during long term cycling.

## Conclusions

In this paper, V-doped LiNi_0.84_Co_0.10_Mn_0.06_O_2_ cathode material was synthesized by solid-state reaction. V-doped NCM samples exhibited better cyclic stability and rate capability (at rates as high as 2 C) compared to pristine NCM. Among them, the 0.005 M vanadium doped NCM showed the excellent structural stability and best electrochemical performances. It can be inferred that the amount of vanadium that can be substituted into the transition metal sites is limited and residual vanadium produces deleterious Li_3_VO_4_ impurity. The introduction of an appropriate amount of vanadium dopant not only provides smooth and rapid lithium ion insertion-extraction by large lattice parameters but also increase the electronic conductivity of NCM. More importantly, it has a positive effect on strong bonding between vanadium and oxygen, enabling remarkable structural stability. As a result, we can conclude that V-0.005 NCM can be regarded as a promising cathode for next-generation LIBs.
